# Towards the Intravascular Delivery of 3D-printed Leached SMP for Endovascular Treatment of Intracranial Aneurysm

**DOI:** 10.1007/s10439-025-03816-w

**Published:** 2025-08-03

**Authors:** Sergio Ruiz Vega, Juan C. Mesa, Coral Blanquer, Cristian Barinaga, Tanner L. Cabaniss, Angel Enriquez, Bradley N. Bohnstedt, Chung-Hao Lee, Hyowon Lee

**Affiliations:** 1 Weldon School of Biomedical Engineering, Purdue University, West Lafayette, IN 47907 USA; 2 Center for Implantable Devices, Purdue University, West Lafayette, IN 47907 USA; 3 Birck Nanotechnology Center, Purdue University, West Lafayette, IN 47907 USA; 4 School of Aerospace and Mechanical Engineering, The University of Oklahoma, Norman, OK 73019-0390 USA; 5 Department of Neurological Surgery, Indiana University School of Medicine, Indianapolis, IN 46202 USA; 6 Biomechanics and Biomaterials Design Laboratory (BBDL), The Bioengineering Department, University of California Riverside, Riverside, CA 92521 USA

**Keywords:** Shape memory polymers (SMPs), Intracranial aneurysm (ICAs), Joule-heating, Electrolytic detachment (ED), Intracranial embolization, Radial compression

## Abstract

**Purpose:**

Intracranial aneurysms (ICAs) pose a serious clinical risk due to their potential for rupture, leading to subarachnoid hemorrhage, high morbidity, and mortality. This study aims to develop a proof-of-concept device for the targeted delivery of shape memory polymers (SMPs)-based embolic devices to improve aneurysm occlusion and reduce recurrence.

**Methods:**

A novel system was designed combining a radial compression fixture and an electronic device for Joule heating and electrolytic detachment (ED). Three SMP geometries (5, 6.5 mm spherical, and patient-specific) were evaluated for the shape recovery and thermal responses. In-vitro testing was performed using 6.5 mm and patient-specific geometries in PDMS aneurysm phantoms under physiological relevant conditions utilizing ovine blood.

**Results:**

Controlled activation of the SMPs at currents of 400 mA achieved reproducible shape recovery ratios (SRRs) up to 75.71%, with detachment occurring at < 100 mA. Surface temperatures remained below 45 °C. In-vitro deployment resulted in aneurysm sac occlusion of 90.32% (patient-specific) and 94.12% (idealized), without evidence of thermal damage or gas accumulation. Flow visualization confirmed reduced bubble entry into the aneurysm sac post-deployment.

**Conclusion:**

This study demonstrates the feasibility of a targeted delivery system for patient-specific ICA treatment using SMPs. While further refinement and in-vivo validation are required, these findings highlight the potential of SMPs as durable embolization devices capable of conforming to complex aneurysm geometries and providing more effective occlusion compared to current methods.

## Introduction

Unruptured intracranial aneurysms (ICAs) are focal dilations of the vascular wall in the brain, that affect approximately 3-5% of the general population [1]. While most ICAs remain asymptomatic, sudden rupture of an ICA can lead to an aneurysmal subarachnoid hemorrhage (aSAH), a life-threatening condition associated with high rates of morbidity and mortality [2]. The annual incidence of aSAH ranges from 10 to 14 per 100,000 people worldwide [3], resulting in severe and even fatal complications. The probability of rupture is influenced by factors such as aneurysm size, location, smoking, gender and patient demographics [4]. The financial burden associated with ICAs is significant, with procedural costs for treatment exceeding $35,000 and total hospitalization expenses potentially reaching up to $100,000 [5].

Current therapeutic options for ICAs include microsurgical clipping and endovascular embolization. Microsurgical clipping involves a craniotomy followed by the placement of a metal clip at the neck of the aneurysm to isolate it from blood circulation and thereby prevent rupture. Since the introduction of Guglielmi detachable coils (GDCs) in 1991 [6, 7], endovascular embolization has become the preferred treatment due to its minimal invasiveness and improved safety profile, particularly in older patients [8, 9]. GDCs are based on two electrochemical principles: electro-thrombosis and electrolysis. The first promotes clot formation in the positively charged coil by attracting negatively charged particles in the bloodstream, while the latter refers to the electrochemical dissolution of the anodic electrode in a conductive media leading to coil detachment. This process is commonly referred to as electrolytic detachment (ED). Modern coils rely on flow disruption and progressive clotting within the aneurysm. The primary goal of these treatments is to achieve complete occlusion of the aneurysm, which is critical for preventing aneurysm rupture.

Despite advancements in this field such as hydrogel-coated coils, stents and flow diverters, achieving complete aneurysm occlusion remains suboptimal (i.e., < 28%), especially for large aneurysms and/or aneurysms with a complex 3D geometry [10]. Studies report unsatisfactory long-term outcomes, including incomplete occlusion, coil compaction, coil migration, and aneurysm recanalization, which increase the risk of aneurysm regrowth or rupture [10, 11]. Wide-neck and irregularly shaped aneurysms are particularly problematic, as current devices often fail to conform adequately to the aneurysm wall or provide sufficient packing density [12]. Flow diverters, while effective for large and fusiform aneurysms, require prolonged dual antiplatelet therapy, which carries risks of bleeding complications [13, 14]. These limitations frequently need retreatment, leading to increased patient morbidity and additional healthcare costs [10, 15].

Addressing these challenges has urged interest in patient-specific endovascular devices. Shape memory polymers (SMPs) have emerged as promising materials due to their unique thermo-mechanical properties, enabling synthesis into complex geometries and packaging into delivery catheters [16]. SMPs can be programmed to respond to external stimuli, such as heat, pH, light or ultrasound, making them ideal for customized medical applications [17]. Our prior work demonstrated the development of SMPs for ICA treatment [18–20], including the use of a 3D-printing leaching method to fabricate patient-specific SMP foams [21]. Nevertheless, a detailed procedure for packaging such SMPs into endovascular catheters and triggering their deployment within aneurysm sacs has yet to be presented.

This study presents a proof-of-concept endovascular platform for the targeted delivery and deployment of our previous developed SMPs [21] for the endovascular treatment of ICAs. The platform integrates a custom 3D-printed radial compression fixture for packaging into 10 Fr (Ø 3.33 mm) catheters, a custom electronic system for current-controlled Joule heating to trigger shape recovery (SR), and an ED mechanism for on-demand release. Three SMP geometries: two spherical designs (5, 6.5 mm) and a patient-specific configuration were evaluated through benchtop testing to assess geometry-dependent performance and then deployed under physiologically relevant conditions *in vitro*. The device achieved reproducible activation below 45 °C with shape recovery ratios (SRRs) up to 75.71%, and complete detachment at currents below 100 mA, without causing potential thermal damage or excessive bubble formation. In-vitro deployment resulted in sac occlusion of 90.32 and 94.12% in patient-specific and idealized phantoms, respectively, and qualitative flow analysis confirmed reduced bubble entry post-deployment. These findings support the feasibility of this minimally invasive, thermally triggered platform and its potential for future clinical applications of our SMP-based embolic devices in ICA treatment.

## Materials and Methods

### Device Design and Fabrication

**Fig. 1:**
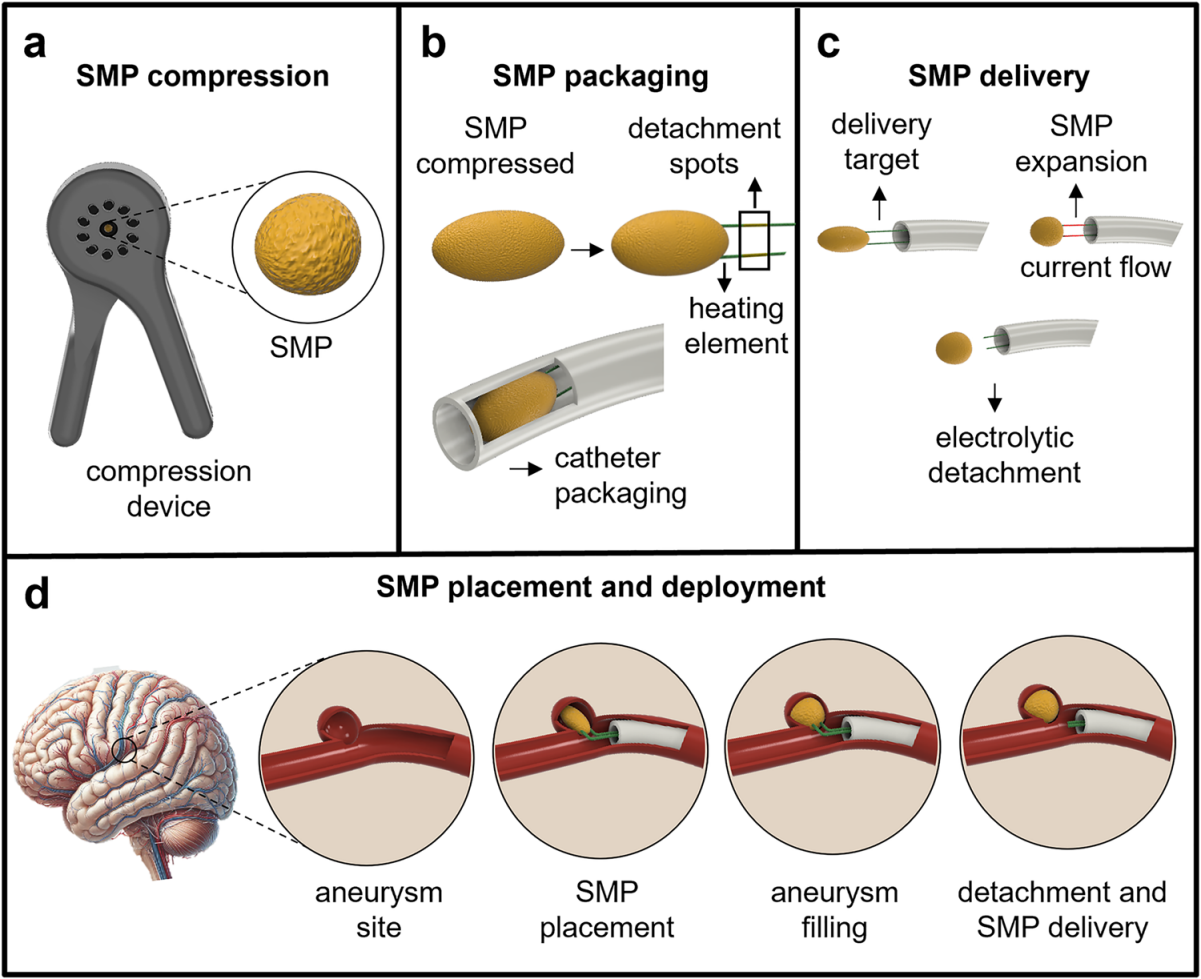
Primary steps for SMP compression, packaging, and delivery: **a** Radial compression of the SMP using our custom 3D-printed hand-held fixture; **b** Packaging of the compressed SMP, highlighted by the inclusion of the heating element, followed by insertion into an endovascular catheter; **c** Delivery of the packaged SMP to the target site through a catheter, followed by heating expansion and detachment of the cable for final delivery into the aneurysm sac. **d** A detailed representation of the steps involved in the placement and deployment of the SMP into the aneurysm sac

Figure 1 illustrates the three stages of our proposed approach: compression, packaging, and delivery. The workflow begins with the compression of the SMP utilizing a custom hand-held 3D-printed fixture (Fig. 1a). Following compression, we integrate the heating element, a polytetrafluoroethylene (PTFE) coated 304 stainless steel (SS) wire 0.127 mm diameter (Component Supply, Inc, Sparta, TN, USA) into the SMP for subsequent packaging into an endovascular catheter (Fig. 1b). Finally, leveraging the capabilities of our electronic device, we expand the SMP via Joule heating in the aneurysm sac and deploy it utilizing ED (Fig. 1c). Figure 1d summarizes the delivery and deployment steps of the SMP into the aneurysm sac.

### Compression Device

**Fig. 2:**
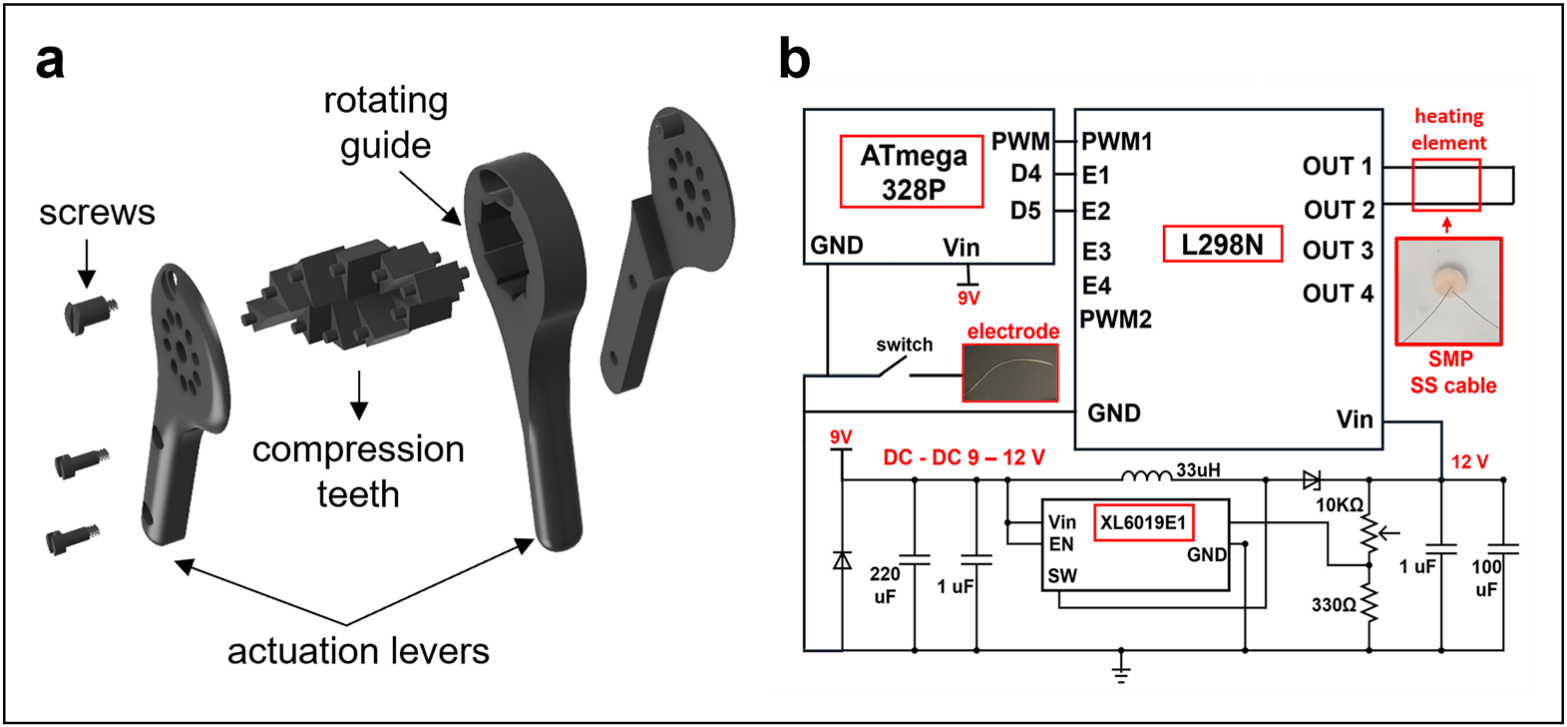
Hand-held compression fixture CAD design and circuit schematic. **a** 3D representation of the main components of the compression fixture, including screws to assemble the components, compression teeth for radial compression, a rotating guide for the teeth, and actuation levers to operate the device. **b** Schematic of the main circuit elements for inducing Joule heating and ED, featuring a 9 V DC source to connected to a DC-DC step-up converter for a 12 V power supply, an L298N H-bridge for power regulation modulated by a PWM signal, an ATmega328P microcontroller, a needle sacrificial electrode for ED, and connections to the SMP and SS heating element

To enable endovascular delivery of the SMP device, we developed a custom-made radial compression tool designed to reduce the device diameter for introduction into a 10 Fr (Ø 3.33 mm) catheter. The design was inspired by commercial stent crimpers, and it features three main components: the compression teeth, a rotating guide, and actuation levers (Fig. 2a), which was developed to maximize the compressive capabilities of the SMP while securing its structural integrity. The device was fabricated using poly-lactic acid (PLA) with an open-source 3D printer (Prusa MK4, Prusa Research, Prague, Czech Republic). In the open position, the compression teeth form a 7 mm diameter circle, accommodating the uncompressed SMP. Upon actuating the levers, the teeth are driven inward to deform the SMP into a cylindrical profile.

### Joule Heating and ED Circuits

Our approach incorporates two mechanisms driven by a custom-made electronic system: Joule heating and ED. To facilitate current flow through the SMP, we used a 15 cm long PTFE-coated SS wire as the heating element, which triggers the SR. We designed a circuit system using conventional electronics to control the current using a pulse-width modulation (PWM) direct current (DC) voltage signal (Fig. 2b). To deliver the necessary current and voltage to drive the system for the heating stage (12 V, 400 mA), we utilized an open-source Dual H Bridge Drive Module (BOJACK L298N Motor DC Dual H-Bridge Motor Driver Controller, Amazon, Inc., Seattle, WA, USA). The power supply consisted of a conventional 9 V Alkaline DC battery (9 Volt Alkaline Everyday Battery, Amazon Inc., Seattle, WA, USA) connected in series with an open-source DC-DC step-up 9–12 V module (XL6019 Step Up DC-DC Power Module, Amazon, Inc., Seattle, WA, USA). An open-source microcontroller (ATmega328P, Microchip Technology, Chandler, AZ, USA) was used to control the PWM signal for current and induce temperature tuning. All electronic components were housed in a 3D-printed PLA case.

To perform the ED, we removed two segments of the PTFE coating on the heating element, each measuring ~ 500 µm in length, thereby exposing the conductive SS wire. This facilitates the electrolysis process, enabling in-situ detachment. We programmed our system to deliver the current in both directions of the heating element to induce ED at the assigned detachment region utilizing a current of ~ 100 mA.

### Compression and Shape Recovery (SR) Assessment

To assess the mechanical reprogramming and thermal SR of SMPs, we employed three geometries: two spherical samples with a diameter of 5 and 6.5 mm, respectively, and a patient-specific configuration with an approximate diameter of 5 mm, characterized by a non-circular cross-section. All SMP samples were fabricated with a 40 and 20% infill density, respectively [21]. Prior to compression, the SMPs were conditioned in an oven at 75 °C for 15 min above the material’s glass transition temperature *T*_*g*_ to induce the rubbery state and enable plastic deformation. The heated/softened SMPs were then shaped using our custom compression fixture and cooled at 4 °C for 10 min to preserve their compressed (reprogrammed) configuration.

To evaluate the device’s ability to trigger SR, nine samples were tested for each geometry (*n* = 3 per thermal condition, total *n* = 27). Samples were randomly assigned to one of the three groups (thermal conditions): (i) Activated group—exposed to a controlled current of 400 mA at 12 V for 4 s using the electronic device, followed by a switch-controlled on–off cycle for up to 60 s; (ii) PBS group—submerged in phosphate-buffered saline (PBS) at 37 °C for 60 s; and (iii) Room temperature group—placed in a petri dish at room temperature for 60 s.

Following treatment, all samples were imaged, and dimensional measurements were made using ImageJ (National Institutes of Health, Bethesda, MD, USA). For spherical geometries, diameters were measured; for the patient-specific design, surface area was used to quantify dimensional changes. In addition, surface temperatures during activation were monitored using an infrared thermal camera (FLIR A325SC, FLIR Systems, Inc., Wilsonville, OR, USA). SRR was quantified utilizing Equation 1, where *A*_*f*_ denotes the final area of the SMP, *Ac* represents the area of the compressed (reprogrammed) SMP, and *A*_*i*_ is the original area of the material prior to compression.

1 $$\text{SRR}\left(\text{\%}\right)=\frac{{A}_{f}-{A}_{c}}{{A}_{i}- {A}_{c}}\times 100\text{\%}$$

Equation 1 SRR percentage estimation considering the final area after expansion (*A*_*f*_), compressed area (*A*_*c*_), and initial area of the material (*A*_*i*_).

### Statistical Analysis

To assess statistical differences in the SRR among the SMP groups and the compression rate across the geometries, we performed a one-way ANOVA test followed by Tukey’s HSD pairwise comparisons with an *α* = 0.05. All analyses were conducted in MATLAB (R2023a, MathWorks, Portola Valley, CA, USA).

### Aneurysm Phantom Fabrication

To replicate physiologically relevant cerebral aneurysm geometries for subsequent in-vitro evaluation for SMP deployment and visualization of flow-blockage performance, two optically clear Polydimethylsiloxane (PDMS) aneurysm phantoms were fabricated: (i) an idealized aneurysm model based on anatomical data from the internal carotid artery combined with average dimensions of human saccular aneurysms [22, 23], and (ii) a patient-specific model derived from anonymized clinical imaging data, previously approved under IRB protocol (University of Oklahoma Health Sciences Center, IRB #7932).

In brief, PDMS (Sylgard 184, Dow, USA) was prepared according to the manufacturer’s instructions at a 10:1 base-to-curing agent ratio. The positive sacrificial core was 3D printed using a desktop fused deposition modeling (FDM) printer (A1 mini, Bambu Labs, China) with polyvinyl alcohol (PVA) filament (Atlas Support, FormFutura, Netherlands), based on the CAD geometry described above. To minimize surface artifacts inherent to layer-by-layer printing, the structure was briefly submerged in room-temperature water for 30 s and then dried in a filament dryer at 60 °C.

**Fig. 3:**
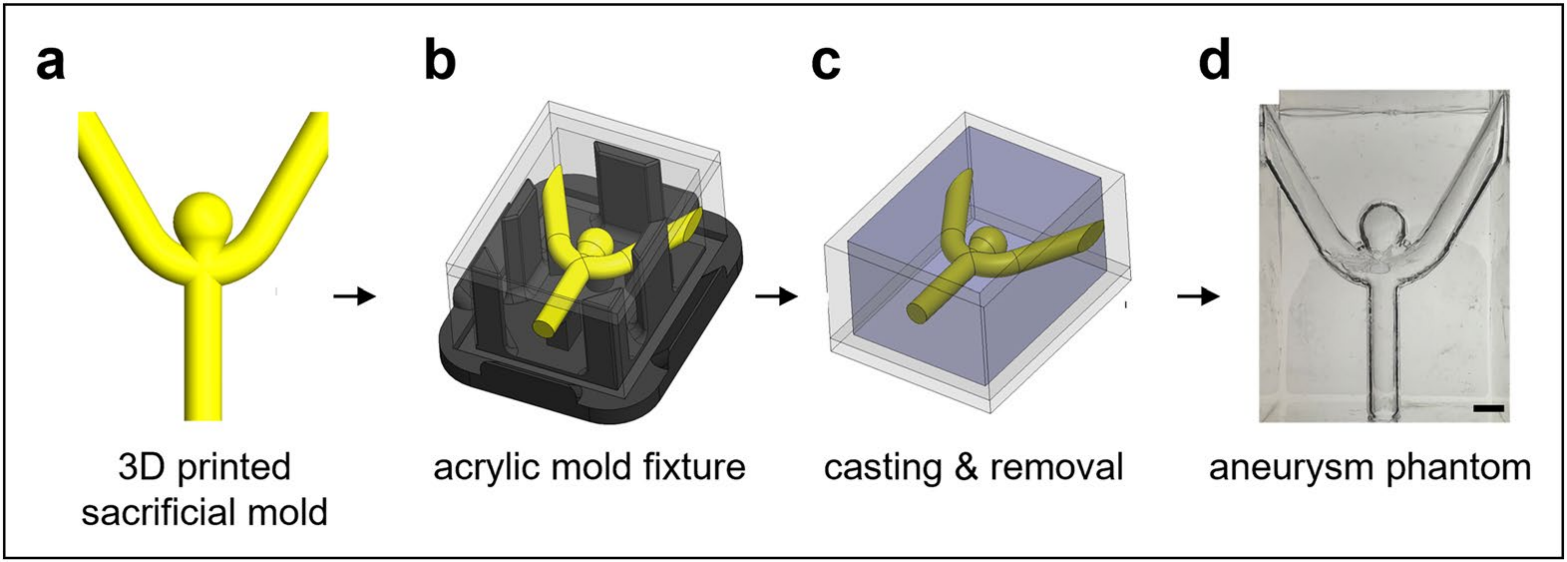
Fabrication process for PDMS aneurysm phantoms. **a** A 3D-printed sacrificial model of an idealized human aneurysm is created using PVA filament via fused deposition modeling. **b** The printed structure is secured within a custom acrylic mold fixture using cyanoacrylate adhesive. **c** The mold is filled with degassed PDMS and cured under vacuum at 60 °C for 12 h. After curing, the PVA insert is dissolved in a 45 °C water bath over 72 h. **d** The resulting transparent PDMS aneurysm phantom preserves the internal vascular geometry. Scale bar = 5 mm

Each printed PVA core was secured within a custom acrylic mold using cyanoacrylate adhesive, after which degassed liquid PDMS was poured into the mold. Degassing was performed in a vacuum oven (BOV-20, Being Scientific, USA) to eliminate trapped air bubbles, and the mold was vacuum-cured at 60 °C for 12 h. Following curing, the acrylic panels were disassembled, and the PDMS block containing the sacrificial core was submerged in a heated, circulating water bath at 45 °C for 72 h to fully dissolve the PVA insert. The completed phantom was subsequently dried under vacuum at 60 °C for an additional 12 h (see Fig. 3 for the details of the fabrications steps).

### Flow Disruption Visualization

To qualitatively evaluate whether SMP deployment disrupted flow entry into the aneurysm sac, we conducted a visualization-based flow analysis using the PDMS aneurysm phantoms described above. SMPs were pre-compressed, positioned within the sac, and activated under the same flow rate and conditions as in the in-vitro blood experiments (see In-Vitro Study for more details). A hydrocarbon mineral oil (Vacuum Pump Oil 20, VWR®, VWR Inc, Radnor, PA, USA) was used as the working fluid due to its optical clarity and ability to support stable visualization of bubble tracers. Air bubbles were introduced upstream to serve as flow indicators, and their movement was recorded following SMP deployment. Frame captures were analyzed using ImageJ to observe whether the flow continued to enter the aneurysm sac after placement, providing visual insight into the potential for flow disruption.

### In-Vitro Study

To evaluate SMP deployment and electrolytic ED to mimic physiological conditions, we conducted in-vitro experiments using the two optically transparent PDMS aneurysm phantoms (ideal carotid artery bifurcation and a patient specific). The SMPs were pre-compressed and delivered into the aneurysm sac catheter, as described previously.

Heparinized ovine blood at 37 °C was circulated at a flow rate of 60 mL/min using a peristaltic pump (Master Flex Model 77201-60, MasterFlex, Gelsenkirchen, Germany). This flow rate was selected to approximate physiological cerebral perfusion. Based on literature values for cerebral blood flow (CBF) ranging from 50 to 60 mL/100 g/min [24, 25], we used an intermediate value of 55 mL/100 g/min to estimate the equivalent perfused tissue mass utilizing Equation 2.

2 $$Tissue \ mass (g) \ =\frac{Flow \ rate (mL/min)}{CBF (mL/100g/min)}\times 100$$

Equation 2 Approximation of the perfused tissue mass for a given flow rate based on cerebral blood flow (CBF).

This calculation yields an estimated perfused brain tissue mass of approximately 110 g, which corresponds to vascular territories typically supplied by small-to-medium cerebral arteries, such as distal branches of the middle cerebral artery (MCA) or the posterior communicating artery [26]. These arteries generally have luminal diameters ranging from 2 to 4 mm [22, 26], consistent with the dimensions of our designed phantoms.

For the Joule heating, the proximal ends of the SMPs’ embedded heating element were connected to the custom-built electronic control unit. A 6 cm platinum (Pt) wire electrode (Pt wire 99.9% 30 Gauge, µGems, Amazon, Inc., Seattle, WA, USA) was used for ED due to its high electrochemical stability, and biocompatibility [27]. To monitor the heating effects on the aneurysm sac and its surroundings, we positioned a Pt 100 Ω thermocouple connected to a digital thermometer (Omega RDXL4SD, Omega Engineering, Inc., Norwalk, CT, USA).

Following SMP expansion, the system automatically sets the Pt electrode to initiate ED. Current was applied through the heating element, targeting the detachment sites. The entire process was continuously recorded using a digital camera for subsequent image analysis to assess SR and ED performance.

## Results

### Temperature Characterization and Safety Assessment

**Fig. 4:**
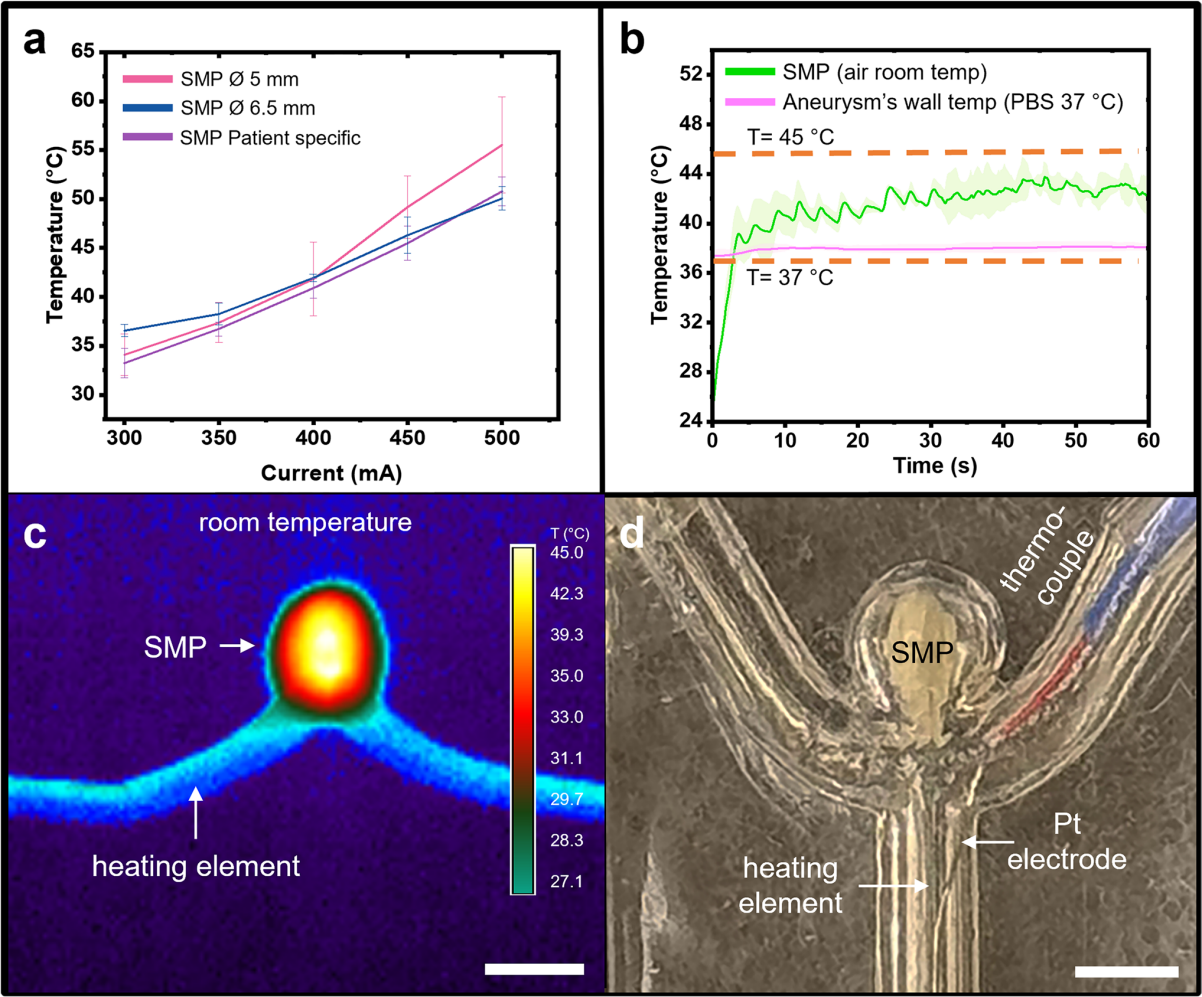
Temperature profile assessment and graphical representation for the SMPs **a** Current and temperature relationship over the SMPs utilizing the electronic system from 300 to 500 mA. **b** Representative temperature profile corresponding to the 5 mm SMP. The green line indicates the surface temperature of the SMP induced by the heating element at room temperature with an average value of approximately 41.42 ± 2.6 1 °C. The purple line represents the aneurysm’s wall temperature measured by a thermocouple in a PBS solution (37 °C), reaching a thermal distribution average of 38.01 ± 0.16 °C. **c** Photograph of the experimental setup for the SMP heating distribution in room temperature air, highlighting the heating element and the heating map around the SMP. **d** A representative photograph of the in-vitro setup for the SMP utilizing the PDMS phantom model, representing an idealized aneurysm from the internal carotid artery, highlighting the expanded SMP, the thermocouple, the heating element, and the Pt electrode. Scale bars = 5 mm

The performance of the Joule heating mechanism was evaluated to assess its effectiveness and safety across the previously defined SMP geometries. As shown in Fig. 4a, all configurations exhibited a consistent increase in surface temperature with increasing current from 300 to 500 mA. A current of 400 mA was identified as the appropriate working point, resulting in average surface temperatures of 41.81 ± 3.75 °C (5 mm sphere), 40.90 ± 2.00 °C (6.5 mm sphere), and 41.95 ± 0.38 °C (patient-specific). These values remained below the 45 °C safety threshold while exceeding the material’s *T*_*g*_*,* enabling controlled and safe shape recovery. This current setting was effective under the tested conditions and geometries, though it may be further tuned based on material properties or application-specific requirements.

To assess thermal performance under physiologically relevant conditions, a bench-top setup was implemented using the 5 mm SMP geometry within the aneurysm phantom based on a carotid artery bifurcation, as previously described in the methodology. As shown in Fig. 4b and a representative surface temperature profile was recorded during controlled current application. The system delivered a 400-mA pulse at 12 V for 4 s, followed by an on–off control strategy to maintain the SMP surface temperature below 45 °C. This approach resulted in an average SMP temperature of 41.42 ± 2.61 °C, while the surrounding aneurysm wall remained near physiological conditions, at 38.01 ± 0.16 °C in PBS maintained at 37 °C.

Fig. 4c presents a thermal image of the SMP during activation, showing a rapid increase from room temperature to approximately 40 °C, followed by stable fluctuations between 40 and 44 °C, confirming that the heating element and control scheme provide reproducible and thermally safe activation. Figure 4d illustrates the bench-top setup used to monitor temperature profiles, highlighting the positioning of the SMP, heating element, a thermocouple and Pt electrode. All temperature assessments for the additional SMP geometries (6.5 mm and patient-specific) were conducted using the final setup for the in-vitro study (see In-Vitro Study for more details) under physiologically relevant conditions. This finding underscores our system’s potential for practical application in endovascular therapies, ensuring that it does not cause harmful thermal damage *in situ*.

### Compression and SR Evaluation

**Fig. 5:**
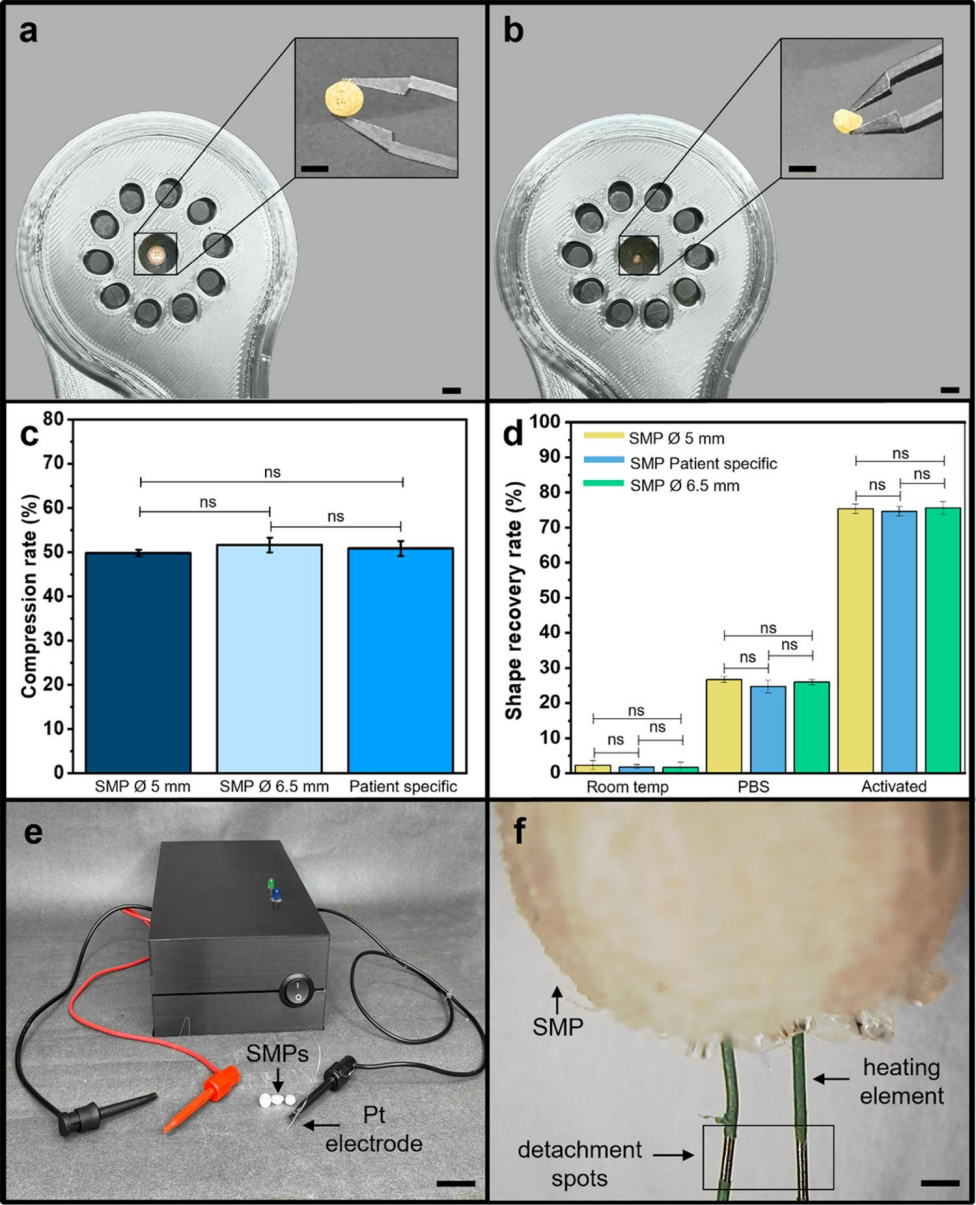
Evaluation of SMPs compression and SR using the proposed device. **a** Representation of Initial positioning of SMP within the compression device after heating to 75 °C. Scale bars = 5 mm. **b** Cylindrical compression of the SMP upon activation of the actuation levers, illustrating the radial compression. Scale bars = 5 mm. **c** Compression rate percentages for the three different tested SMP geometries. **d** SRR percentages of the SMPs triggered by the device, PBS at 37 °C, and room temperature. **e** Integrated device setup, highlighting the electronics housing connected to the heating element embedded in the SMP, and the Pt electrode. Scale bar = 2 cm. **f** A zoomed-in view of the delivered SMP, highlighting the detachment spots and the PTFE-coated heating wire. Scale bar = 500 µm

To evaluate the mechanical and thermal behavior of the SMPs, each sample was heated above its *T*_*g*_ and compressed using the handheld device (Fig. 5a), resulting in uniform cylindrical deformation as shown in Fig. 5b.

Figure 5c presents the calculated compression rates for the three geometries: 49.81 ± 0.75% (5 mm sphere), 51.68 ± 1.68% (6.5 mm sphere), and 50.85 ± 1.71% (patient-specific). The initial diameters for the SMPs were 5.08 ± 0.20 mm (5 mm sphere group) and 6.45 ± 0.09 mm (6.5 mm sphere group). For the patient-specific group, which lacked a circular cross-section, the initial geometry was characterized by area: 22.22 ± 0.65 mm^2^. No statistically significant differences in compression rate were found among geometries (all *p*-values > 0.05), indicating consistent compressibility across designs.

Figure 5d shows the SRR across different thermal conditions. Under heating-based activation (Activated), SRRs were 75.42 ± 1.36% (5 mm sphere), 74.71 ± 1.22% (6.5 mm sphere), and 75.66 ± 1.76% (patient-specific). In contrast, SMPs exposed to PBS at 37 °C achieved SRRs of 26.68 ± 0.84%, 24.76 ± 1.71%, and 26.00 ± 0.69%, while those at room temperature exhibited minimal recovery: 2.37 ± 1.26%, 1.88 ± 0.61%, and 1.67 ± 1.43%, respectively. No statistically significant differences in SRR were observed among geometries within any activation condition (all *p*-values > 0.05), suggesting that the recovery performance was geometry-independent for the utilized conditions.

Finally, Fig. 5e illustrates the integrated device used for SMP activation, including the electronic control box, Pt electrode, and heating element, with detachment spots highlighted. A close-up of the ED deployment is shown in Fig. 5f.

### Flow Disruption Visualization

**Fig. 6:**
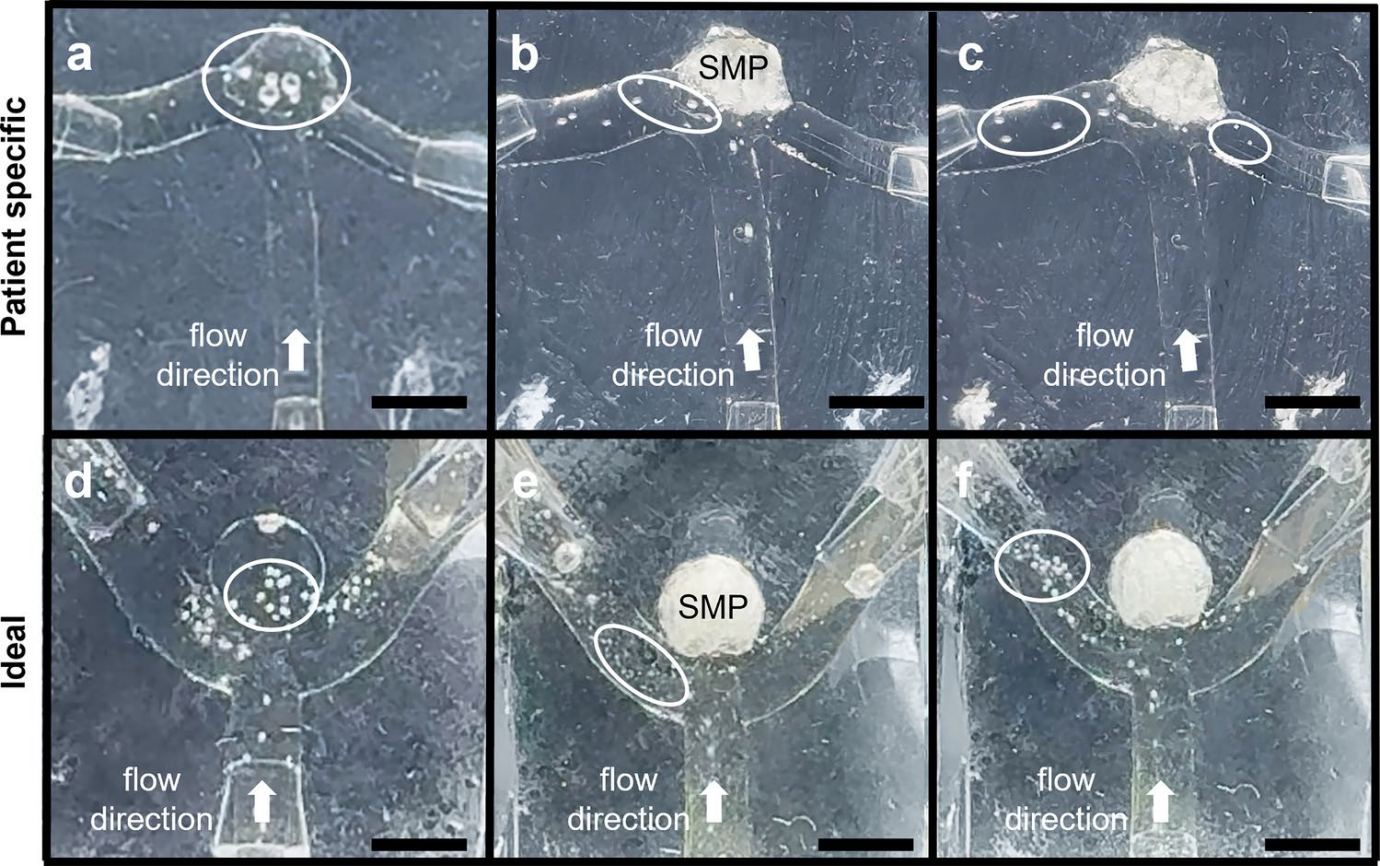
Qualitative visualization of flow disruption by SMP devices in idealized and patient-specific aneurysm models. **a** Bubble accumulation within the aneurysm sac prior to SMP deployment in the patient-specific model. **b** Early-stage dispersion of bubbles immediately following SMP deployment, indicating partial flow interference. **c** Later-stage visualization showing sustained bubble diversion and flow disruption. **d** Pre-deployment bubble distribution within the aneurysm sac for the idealized phantom model. **e** Shortly after SMP deployment, bubbles begin to divert away from the aneurysm neck, indicating early flow disruption. **f** Further downstream stabilization and sustained bubble redirection, demonstrating partial occlusion and flow disruption. Scale bars = 5 mm

Qualitative flow analysis revealed distinct changes in flow inside the aneurysm sac following SMP deployment in both patient-specific and idealized PDMS phantoms (Fig. 6). Prior to deployment, tracer bubbles consistently entered the aneurysm sac, indicating unblocked flow access (Fig. 6a and d). Upon SMP placement and expansion (Fig. 6b and e), a visible reduction in bubble penetration into the sac was noted. After full deployment and electrolytic detachment (Fig. 6c and f), tracer bubbles were primarily redirected along the parent artery, with minimal or no entry into the aneurysm cavity. These results demonstrate that the expanded SMP device substantially altered flow trajectories and partially occluded inflow into the sac. Although this is a preliminary qualitative approach, the observed flow redirection suggests that the SMP structure functioned as a mechanical barrier to intra-aneurysmal circulation. This preliminary evidence supports the device’s capacity to place and deliver the SMPs into the aneurysm sac to promote localized flow disruption, an essential mechanism in endovascular therapy.

### In-Vitro Study

**Fig. 7:**
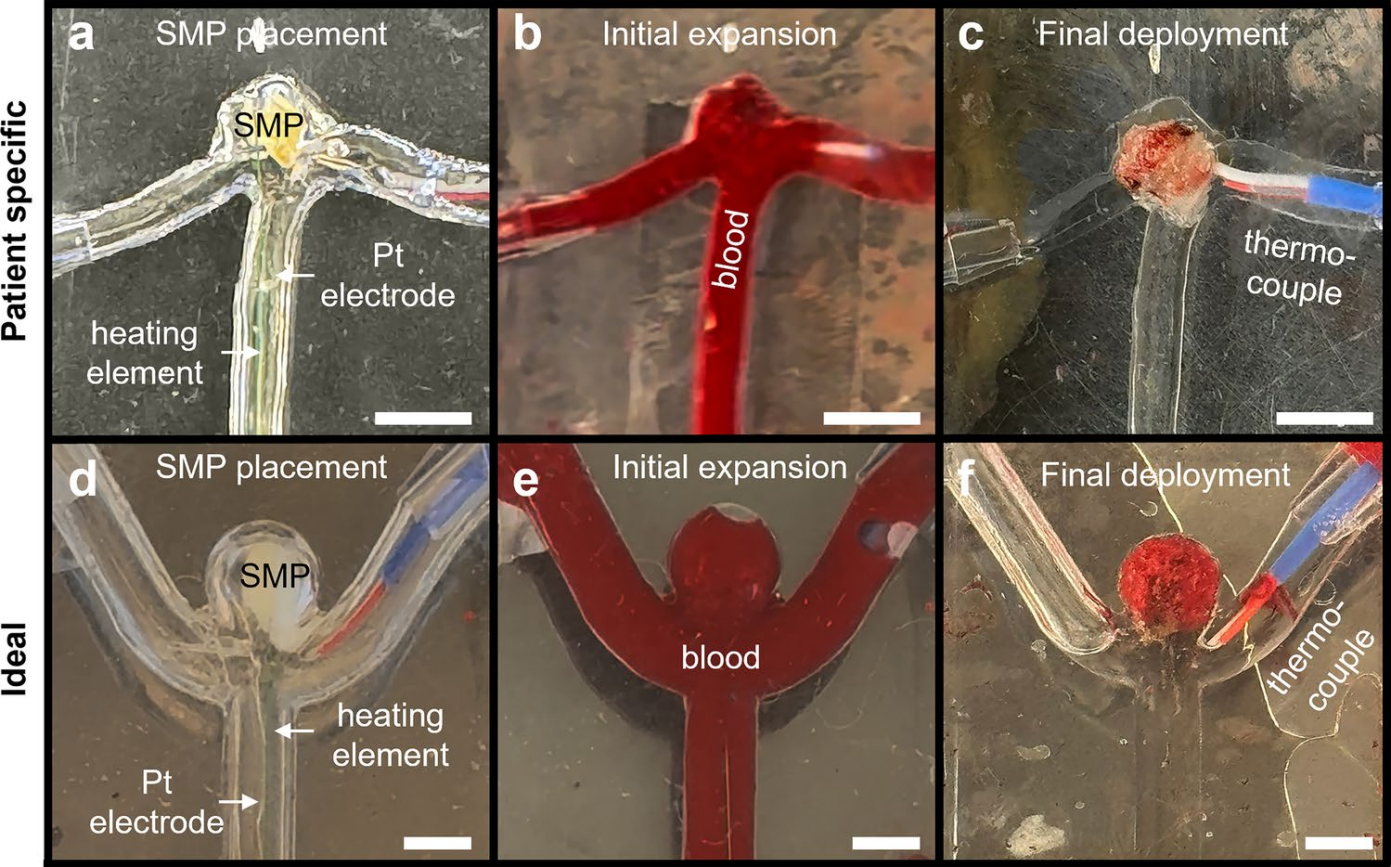
In-vitro deployment of SMPs in patient-specific and idealized carotid artery PDMS aneurysm phantoms. **a** Placement of the compressed SMP for the patient-specific model, highlighting the Pt electrode and heating element prior to blood flow initiation. **b** Initial expansion of the SMP triggered by Joule heating during physiological flow across the phantom. **c** Final placement and full expansion of the SMP within the aneurysm sac following electrolytic detachment. **d** Placement of the compressed SMP into the sac region of the idealized bifurcation model, with the Pt electrode and heating element aligned for activation. **e** Initial expansion of the SMP after blood flow initiation and thermal activation. **f** Final configuration after full deployment and detachment, showing the expanded SMP occupying the aneurysm sac and the thermocouple positioned for temperature monitoring. All deployments were completed below 40 °C with detachment currents < 100 mA, resulting in aneurysm sac occlusion of 90.32 and 94.12% in patient-specific and idealized phantoms, respectively. Scale bars = 5 mm

Figure 7 showcases the in-vitro deployment of the SMP device in both patient-specific (Fig. 7a–c) and idealized bifurcation (Fig. 7d–f) PDMS aneurysm phantoms. In the patient-specific model, the SMP was positioned within the aneurysm sac (Fig. 7a) and expanded upon Joule heating after flow initiation (Fig. 7b) as explained above. The average temperature measured at the aneurysm’ activation site was 37.91 ± 2.42 °C, and electrolytic detachment was achieved with currents consistently below 100 mA. Complete detachment occurred within 180.05 ± 13.08 s without evidence of gas accumulation or prominent bubble formation. After flushing the phantom, post-deployment evaluation revealed that the SMP occupied 90.32% of the aneurysm sac (Fig. 7c), indicating substantial volumetric occlusion.

On the other hand, for the idealized model a similar sequence was evidenced (Fig. 7d–f). The SMP was placed in the sac (Fig. 7d), activated to induce shape recovery (Fig. 7e), and successfully detached following full expansion (Fig. 7f). The average temperature during actuation was 38.02 ± 2.42 °C, with no observable signs of thermal damage. Occlusion analysis showed that the device occupied 94.12% of the aneurysm sac. Together, these results demonstrate consistent device performance across anatomical configurations and support the generalizability of our SMP-based approach for the treatment of endovascular occlusion. While these findings serve as a proof-of-concept, further validation in more physiologically complex and clinically relevant in-vivo models will be necessary to fully assess the long-term efficacy and safety of this envisioned technology.

## Discussion

SMPs have shown promising potential for the treatment of ICAs due to their thermo-mechanical properties, enabling a more personalized approach for complex aneurysm geometries. We demonstrated that the delivery and deployment of SMPs into both idealized and patient-specific in-vitro models under physiologically relevant conditions resulted in aneurysm sac occlusion > 90% within 180 s (Fig. 7), supported by flow visualization indicating redirected flow and limited bubble entry (Fig. 6). While the flow assessment was qualitative, occlusion outcomes were consistent across models. These preliminary results suggest that successful sac filling can be achieved through proper implementation and positional deployment of our device. Future computational fluid dynamics (CFD) analysis will be essential to quantitatively assess flow disruption effects and further refine the system for translational applications.

This study presents a proof-of-concept platform that integrates packaging, delivery, and deployment of previously developed SMPs into both idealized carotid and patient-specific aneurysm phantoms. By leveraging conventional electronics to induce Joule heating and electrolytic detachment, alongside a custom radial compression fixture, we demonstrated deployment across three different geometries, including a patient-specific model. Unlike prior studies focused primarily on material characterizations, our work integrates the full delivery mechanism, showcasing reliable deployment and post-deployment sac occlusion. To our knowledge, this is the *first-of-its-kind* system that combines both Joule heating and ED for SMP deployment in the endovascular treatment of ICAs. A comparative summary of SMP-based aneurysm devices, actuation methods, and delivery strategies is provided in Table 1.

**Table 1:** Comparison of the state-of-the-art SMP systems for the treatment of ICAs

References	SMP application brief description	Triggering mechanism	Validation method (Animal/Human In vivo studies, in vitro, benchtop	Commercial vs. research
This work	A highly porous SMP, fabricated through a 3D-printing and leaching method, integrated with a compression and electronic device for packaging and delivery into the aneurysm sac	Joule heating is achieved through a specialized electronic device. The system is also equipped with a handheld compression fixture and module for ED.	In-vitro assessment using carotid ideal and patient specific phantom aneurysm models for placement and deployment of SMPs leveraging joule heating and ED	Research
[28]	An SMP PU foam polymer that is crimped onto a Pt-tungsten coil for catheter delivery, which self-expands to a predetermined volume upon contact with blood.	Self-expansion upon contact with blood. The device includes an external apparatus for coil detachment.	Animal testing was performed by [29] Human studies conducted by [30]	Commercial (Trellix Embolic Coil ®, Shape Memory Medical, Santa Clara, CA, USA), EU (CE) clearance Annex II Registration #: 530394 [28]
[31]	An EVOH copolymer that provides complete filling and distal penetration of peripheral lesions. The polymeric matrix contains DMSO and micronized Ta powder to provide contrast visualization under fluoroscopy.	When in contact with external fluids, the pre-mixed polymeric solution induces solidification *in situ*. Due to the Ta particles, the polymer has contrast for visualization	Animal testing performed by [32] Human studies conducted by [32–34]	Commercial (Onyx^TM^, Medtronic, Minneapolis, MI, USA) (FDA PMA#: P030004) [35]
[36]	PU SMPs with 4% filling of tungsten before curing, triggering radio-opacity properties for imaging and safe delivery to the aneurysm site.	Heat, yet authors did not delve into details about this mechanism.	A porcine model to determine the opacity ratio of the SMP, revealing high visibility under conventional imaging techniques after implantation in-situ.	Research
[18]	An aliphatic urethane-based SMP with temperature threshold activation within safe rates for an in-vivo application.	Temperature ratings (T > 37 °C) via an external mechanism.	Benchtop assessment only for the SMP properties without in-vitro or in-vivo studies.	Research
[20]	A PU foam infiltered with conductive CNTs to facilitate heat conductivity and tunable resistivity.	Joule heating via a conventional DC power supply through a conductive wire with current ratings up to 1.25 A.	An in-vitro assessment with a simple aneurysm model.	Research
[37]	A solid PU tube SMP stent fabricated by dip coating of SS laser-etched mesh pattern for the treatment of arterial stenosis (vessels’ narrowing).	Photothermal optical actuation utilizing a laser with power ratings of 8W approximately.	In-vitro assessment in artery model of 4 mm diameter, revealing promising outcomes for SMP expansion (no consideration of physiological flow).	Research
[38]	An SMP foam-over-wire embolization device equipped with a crafted laser heating system for delivery and placement in-situ.	Laser heat induction through a crafted optical system with temperature ratings of ~ 50 °C.	In-vitro and in-vivo evaluation via a porcine saccular aneurysm model, achieving implantation with a theoretical volume occlusion of ~ 72%.	Research
[39]	A PU-based SMP foam based on the H60 chemistry fabrication technique was reported with diameter sizes ranging from 8 to 12 mm.	Expansion upon contact with blood was observed; however, the precise triggering mechanism remains unspecified in detail.	In-vivo porcine assessment focused primarily on healing and biocompatibility, revealing minimal inflammatory response (90 days post-implantation).	Research

*CNT* carbon nanotubes, *DMSO* dimethyl-sulfoxide, *EVOH* ethylene vinyl alcohol copolymer, *PU* polyurethane, *Ta* tantalum

The compression fixture developed here enables a size reduction of the SMPs for delivery through a 10 Fr (Ø 3.33 mm) catheter. Our current system achieves approximately 50%-dimensional reduction (see Fig. 5c) across the studied geometries. While all compressed diameters can be packed into the 10 Fr catheter, we acknowledge the need of further enhancement to compression fixture to meet the constraints of standard clinical catheters (<6 Fr, Ø 2 mm) for ICA treatment [40]. A potential solution may involve redesigning the fixture to incorporate additional compression teeth or a multi-stage compression sequence to enable a more uniform and higher compaction.

The custom electronic system leverages Joule heating using a current-controlled strategy, maintaining SMP temperatures below 45 °C while achieving SRRs up to 75.71% across all geometries (Fig. 5d). This represents an improvement over our previous work, where SRRs were limited nearly to 50% under similar thermal conditions [21]. Importantly, the current was applied exclusively through the SMP structure, avoiding direct exposure to tissue or blood. The electronics support tunable control of current based on SMP properties, providing flexibility for optimizing shape recovery without exceeding thermal safety thresholds.

Compared to the earlier efforts using carbon nanotube-doped SMPs requiring > 1 A for actuation [20], our system operates at significantly lower currents approximately 400 mA for expansion and under ~ 100 mA for ED, minimizing the risk of thermal damage. No visible thermal injury or excessive gas formation was observed in the phantom models. The addition of a Pt electrode for detachment further reduced bubble formation, a common complication in electrolytic systems, thereby enhancing procedural safety.

Minimizing surgical time is another critical consideration for ICAs interventions, as longer procedures are associated with increased risks and healthcare costs. Standard GDC-based coil embolization can take 30–158 min depending on patient factors and surgical complexity [41], while microvascular clipping may require 3–5 h including imaging and craniotomy preparation [42]. In contrast, our SMP device achieves deployment and sac occlusion within minutes, presenting a faster alternative to coil-packing, which often involves iterative deployment to achieve adequate packing density. Furthermore, while some SMP-based systems avoid external triggers [31, 39], they risk premature expansion upon blood contact. Our externally triggered approach provides temperature control, reducing such complications. Although in-vivo validation is still required, these findings underscore the potential of our minimally invasive and rapid method as an effective treatment strategy for ICAs.

For clinical translation, several limitations must be addressed. Incorporating visualization traces to enable visualization under standard imaging techniques during navigation and deployment. Radiopaque particles into the SMP formulation can be exploited to address this limitation. The compression fixture requires further optimization to pack the device within commercial catheters < 6 Fr. While the current controller is already tunable, further refinement will support dynamic adjustment based on specific SMP properties. These adaptations will be essential for future in-vivo assessments, particularly using rabbit models, which is known as an established platform for ICAs studies [43]. These next steps will be critical to overcoming current limitations and translating this promising technology toward clinical applications for cerebral aneurysms and other vascular disorders.

## Data Availability

The data supporting the findings of this study will be available from the corresponding author upon request.
